# Cholinergic stimulation with pyridostigmine modulates a heart-spleen axis after acute myocardial infarction in spontaneous hypertensive rats

**DOI:** 10.1038/s41598-021-89104-8

**Published:** 2021-05-05

**Authors:** Robson Luiz Bandoni, Pamela Nithzi Bricher Choque, Humberto Dellê, Tercio Lemos de Moraes, Maria Helena Mattos Porter, Bruno Durante da Silva, Gizele Alves Neves, Maria-Claudia Irigoyen, Kátia De Angelis, Valentin A. Pavlov, Luis Ulloa, Fernanda Marciano Consolim-Colombo

**Affiliations:** 1 Biotechnology Laboratory, Postgraduate Program in Medicine, Universidade Nove de Julho (UNINOVE), São Paulo, SP Brazil; 2 Hypertension Unit, Heart Institute (INCOR), Medical School of University of São Paulo, São Paulo, SP Brazil; 3 Departament of Physiology, Federal University of São Paulo (UNIFESP), São Paulo, SP Brazil; 4 Feinstein Institutes for Medical Research, Northwell Health, Manhasset, NY USA; 5 Department of Anesthesiology, Duke University Medical Center, Durham, NC USA

**Keywords:** Cell biology, Immunology, Physiology, Cardiology, Diseases, Risk factors

## Abstract

The mechanisms regulating immune cells recruitment into the heart during healing after an acute myocardial infarction (AMI) have major clinical implications. We investigated whether cholinergic stimulation with pyridostigmine, a cholinesterase inhibitor, modulates heart and spleen immune responses and cardiac remodeling after AMI in spontaneous hypertensive rats (SHRs). Male adult SHRs underwent sham surgery or ligation of the left coronary artery and were randomly allocated to remain untreated or to pyridostigmine treatment (40 mg/kg once a day by gavage). Blood pressure and heart rate variability were determined, and echocardiography was performed at day six after MI. The heart and spleen were processed for immunohistochemistry cellular analyses (CD3^+^ and CD4^+^ lymphocytes, and CD68^+^ and CD206^+^ macrophages), and TNF levels were determined at day seven after MI. Pyridostigmine treatment increased the parasympathetic tone and T CD4^+^ lymphocytes in the myocardium, but lowered M1/M2 macrophage ratio towards an anti-inflammatory profile that was associated with decreased TNF levels in the heart and spleen. Treatment with this cholinergic agent improved heart remodeling manifested by lower ventricular diameters and better functional parameters. In summary, cholinergic stimulation by pyridostigmine enhances the parasympathetic tone and induces anti-inflammatory responses in the heart and spleen fostering cardiac recovery after AMI in SHRs.

## Introduction

Acute myocardial infarction (AMI) triggers a sterile inflammatory response characterized by the recruitment and activation of innate and adaptive immune cells to repair tissue damage^1–3^. AMI also elicits systemic inflammatory responses that result in organism-wide complications reminiscent of that found in sepsis^4–6^. In rodents, AMI triggers hematopoiesis inducing the production of innate immune cells and recruitment of neutrophils and inflammatory monocytes in the damaged tissue^7^. These mechanisms regulating monocyte recruitment from the bone marrow and spleen into the heart during healing have major clinical implications to design novel therapeutic strategies^8, 9^. In addition to innate cells, adaptive immune responses also play a fundamental role in cardiac remodeling after AMI^9^. More specifically, CD4+ T lymphocytes are required for proper healing and might prevent chronic remodeling after AMI. Several studies have shown that CD4+ T regulatory cells (Treg) can modulate inflammation in the myocardium after an ischemic injury^10, 11^. Patients with acute coronary syndrome show infiltration of CD4+ and CD8+ lymphocytes in MI and non-MI areas, and reduced circulating Treg cells with compromised modulatory function^12^. These results suggest systemic mechanisms coordinating the autonomic nervous system, bone marrow, and spleen to modulate the immune response from the atherosclerotic plaque to infarcted myocardium^13^.

Recently, autonomic neural regulation of the immune system has attracted investigators' attention as a novel therapeutic strategy to control inflammation in multiple clinical settings, including myocardial ischemia–reperfusion injury^14^ and MI^15, 16^. The main nerve of the parasympathetic part of the autonomic nervous system—the vagus nerve modulates the immune function by regulating innate and acquired immune cell-mediated responses^17, 18^. Electrical vagus nerve stimulation suppresses aberrant systemic inflammation and decreases serum TNF levels by inhibiting its production in the spleen^19^. The vagus nerve interacts with the splenic nerve that releases norepinephrine in the spleen^20, 21^. Norepinephrine activates β2-adrenoceptors on splenic T lymphocytes that contain the enzyme choline acetyltransferase (ChAT), which synthesizes acetylcholine^22–24^. Vagus nerve stimulation causes an increase in splenic acetylcholine levels. In turn, lymphocyte-derived acetylcholine binds to the α7-nicotinic acetylcholine receptors (α7nAChR) on splenic macrophages to inhibit TNF production^19, 24, 25^. On the other hand, sympathetic nerve hyperactivity in the spleen has been linked to chronic immune-mediated inflammatory diseases^26^. Moreover, central nervous system angiotensin II infusion and hyperthermia are associated with enhanced level of efferent splenic sympathetic nerve discharge and splenic pro-inflammatory cytokine gene expression in rats^27^. These findings show the potential of the autonomic nervous system to regulate immune responses and inflammation; in this regulation the vagus nerve plays a specific role in orchestrating cellular and cytokine immune responses directed towards promoting tissue healing.

Pyridostigmine is a cholinesterase inhibitor and a cholinergic drug used to treat myasthenia gravis, a chronic autoimmune, neuromuscular disease that causes muscle weakness^28^. Pyridostigmine prevents acetylcholine hydrolysis and enhances parasympathetic modulation in normotensive rats^29^. We have previously reported that pyridostigmine treatment prevents deleterious inflammation and oxidative stress in the ischemic myocardium of these rats^30^. Pyridostigmine treatment also increased the proportion of T regulatory cells in peripheral circulation, and decreased activated CD8+ lymphocytes in the spleen. These results suggest that pyridostigmine modulates the immune cell response by regulating splenic lymphocytes^31^. However, the effects of pyridostigmine in infarcted spontaneous hypertensive rats (SHRs) that exhibit autonomic dysfunction and chronic inflammation and may represent an animal model that better mimics the clinical profile of patients with AMI remained unknown. In the present study, we investigated whether pyridostigmine affects splenic lymphocytes and pro-inflammatory cytokines, and its potential to modulate inflammatory, structural, and functional responses after myocardial infarction in SHRs.

## Results

### Pyridostigmine treatment enhances hemodynamic and autonomic parameters

**Figure 1:**
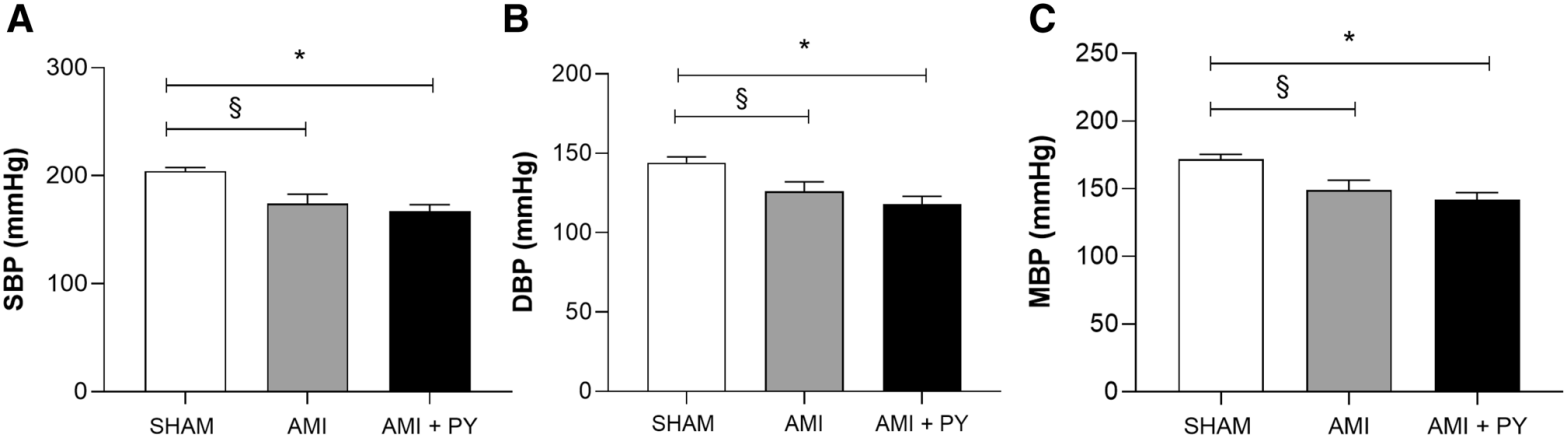
Hemodynamic parameters. (**A**) SBP; Systolic Blood Pressure. (**B**) DBP; Diastolic Blood Pressure, and (**C**) MBP; Mean Blood Pressure; HR: Heart rate. *p < 0.05 versus Sham; ^**§**^*p* < 0.05 versus AMI. Values expressed as mean ± SEM. Statistical significance was determined by one-way ANOVA followed by a multiple comparisons test Tukey’s was performed using GraphPad Prism version 9.0.1. www.graphpad.com.

**Figure 2:**
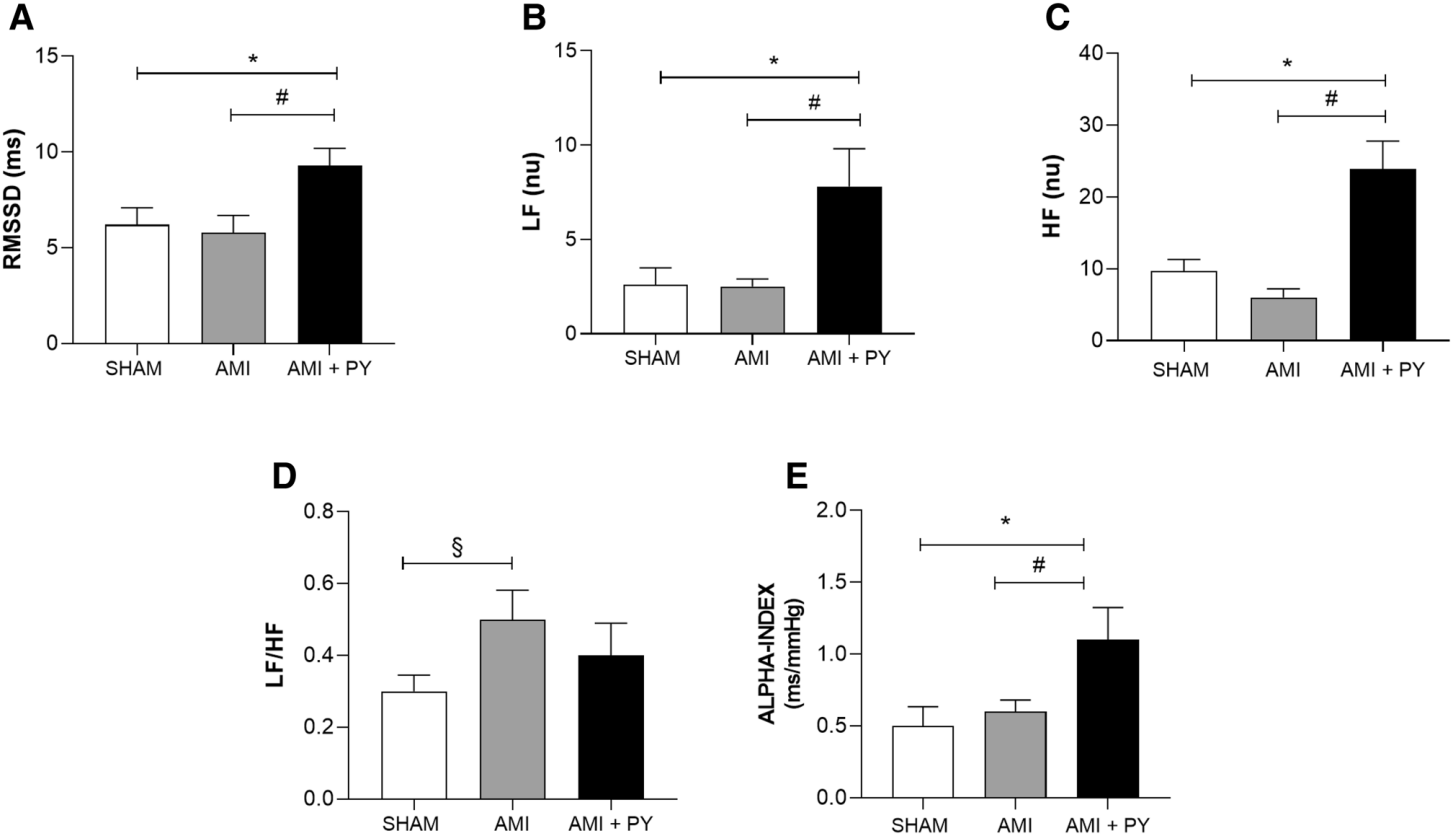
Heart rate variability (HRV) and Baroreflex Sensitivity (alpha-index). (**A**) RMSSD: square root of the mean of the sum of squares of the differences between successive pulse interval values. (**B**) LF: Low Frequency nu: Normalized units. (**C**) HF nu: high frequency in normalized units and. (**D**) LF/HF (**E**) ALPHA-INDEX: baroreflex sensitivity. *P < 0.05 versus Sham; ^#^*P* < 0.05 versus AMI and ^**§**^*p* < 0.05 versus AMI. Values expressed as mean ± SEM. Statistical significance was determined by one-way ANOVA followed by a multiple comparisons test Tukey’s was performed using GraphPad Prism version 9.0.1. www.graphpad.com.

First, we analyzed the hemodynamic variables as presented in Table 1. Sham animals had very high blood pressure (204/144 mmHg) characteristic of spontaneous hypertensive SHRs. MI significantly lowered (*p* < 0.05) systolic (SBP), diastolic (DBP), and mean (MBP) blood pressure as compared to sham animals (Fig. 1). These results indicate decreased cardiac output potentially related to reduced left ventricular contraction caused by ischemic injury. Pyridostigmine treatment affected neither of these hemodynamic parameters. Moreover, all groups had similar heart rate (*p* > 0.05). We also analyzed the autonomic tone via heart rate variability (HRV) and baroreflex sensitivity to determine whether pyridostigmine enhances the parasympathetic modulation (Table 2, Fig. 2). Root mean square of successive differences (RMSSD) reflects the beat-to-beat variance in heart rate and is the primary time domain measurement used to estimate vagal mediated changes reflected in HRV. The RMSSD correlates with HF and reflects self-regulatory capacity. Pyridostigmine treatment significantly increased RMSSD showing its potential to induce cholinergic stimulation (Fig. 2A).

**Table 1:** Hemodynamic parameters in all three groups.

	Sham (n = 5)	AMI (n = 5)	AMI + PY (n = 5)
SBP (mmHg)	204 ± 8.2	174 ± 21.6^§^	167 ± 13.5*
DBP (mmHg)	144 ± 8.4	126 ± 14.8^§^	118 ± 10.9*
MBP (mmHg)	172 ± 8.1	149 ± 17.8^§^	142 ± 11.6*
HR (bpm)	376 ± 28.1	392 ± 27.9	369 ± 39.6*

Values expressed as mean ± SEM*.* ^§^AMI versus Sham; *AMI + PY versus Sham. Values expressed as mean ± standard deviation. Sham: Control Group; AMI: Untreated Infarcted Group; AMI + PY: Infarcted Group Treated with Pyridostigmine SBP: Systolic Blood Pressure; DBP; Diastolic Blood Pressure, MBP = Mean Blood Pressure, HR: Heart rate. *p < 0,05; ^§^*p* < 0,05. Statistical significance was determined by one-way ANOVA followed by a multiple comparisons test Tukey’s was performed using GraphPad Prism version 9.0.1. www.graphpad.com.

**Table 2:** Heart rate variability (HRV) and Baroreflex Sensitivity (alpha-index) in all groups.

	Sham (n = 5)	AMI (n = 6)	AMI + PY (n = 5)
**HRV**			
RMSSD (ms)	6.2 ± 2.0	5.8 ± 2.2	9.3 ± 2.0 *^#^
PIVAR (ms^2^)	28.5 ± 13.0	15.8 ± 6.0	63 ± 28.1 *^#^
LF (nu)	2.6 ± 2.0	2.5 ± 1.0	7.8 ± 4.5 *^#^
HF (nu)	9.7 ± 3.6	6.0 ± 3.0	23.9 ± 8.7 *^#^
LF/HF	0.3 ± 0.1	0.5 ± 0.2 ^**§**^	0.4 ± 0.2
ALPHA-INDEX (ms/mmHg)	0.5 ± 0.3	0.6 ± 0.2	1.1 ± 0.5 *^#^

Values expressed as mean ± SEM. ^**§**^AMI vs Sham; *AMI + PY vs Sham and ^#^AMI + PY vs AMI. HRV: heart rate variability; RMSSD: square root of the mean of the sum of squares of the differences between successive pulse interval values; PIVAR: pulse interval variance; LF: Low Frequency nu: Normalized units; HF nu: high frequency in normalized units; LF/HF: autonomic balance. *P < 0.05; ^#^*P* < 0.05; ^**§**^*P* < 0.05. Statistical significance was determined by one-way ANOVA followed by a multiple comparisons test Tukey’s was performed using GraphPad Prism version 9.0.1. www.graphpad.com.

We also performed the spectral analysis of HRV with the three main spectral components distinguished in short-term recordings: very low frequency (VLF), low frequency (LF), and high frequency (HF). The distribution of the power and the central frequency of LF and HF are not fixed but may vary in relation to changes in autonomic modulations of the heart period. We analyzed LF (Fig. 2B) and HF in both absolute (ms^2^) and normalized units (n.u), representing sympathetic and parasympathetic modulation, respectively. Normalization tends to minimize the effects of LF and HF in total power and emphasizes the balanced behavior (LF/HF) of the two branches of the autonomic nervous system^15, 24, 25^). These results showed that AMI decreased HF (nu) (Fig. 2C), and thereby significantly increased the LF/HF ratio (Fig. 2D) consistent with enhanced sympathetic modulation. Conversely, pyridostigmine treatment significantly increased HF (nu) (Fig. 2C), and decreased LF/HF ratio (Fig. 2D). These results concur in showing pyridostigmine potential to enhance the parasympathetic vagal tone. Alterations of the baroreceptor-heart rate reflex contribute to reduced parasympathetic activity and increased sympathetic activity during cardiovascular diseases, including AMI. This baroreflex sensitivity measured by spectral methods assesses the relationship (in terms of gain) between specific oscillatory components of the two signals. Spontaneous oscillations in blood pressure elicit an oscillation at the same frequency in RR interval by the arterial baroreflex activity. BRS is computed as the average value of the transfer function modulus (i.e., the gain) between systolic pressure and RR interval in the frequency range 0.07–0.14 Hz. The analysis of BRS can provide prognostic information in experimental and clinical studies^32^. We observed that pyridostigmine treatment significantly increased BRS (α-index) indicating an improvement of baroreflex sensitivity (Fig. 2E).

### Pyridostigmine treatment improves cardiac morphofunctional analyzes

**Figure 3:**
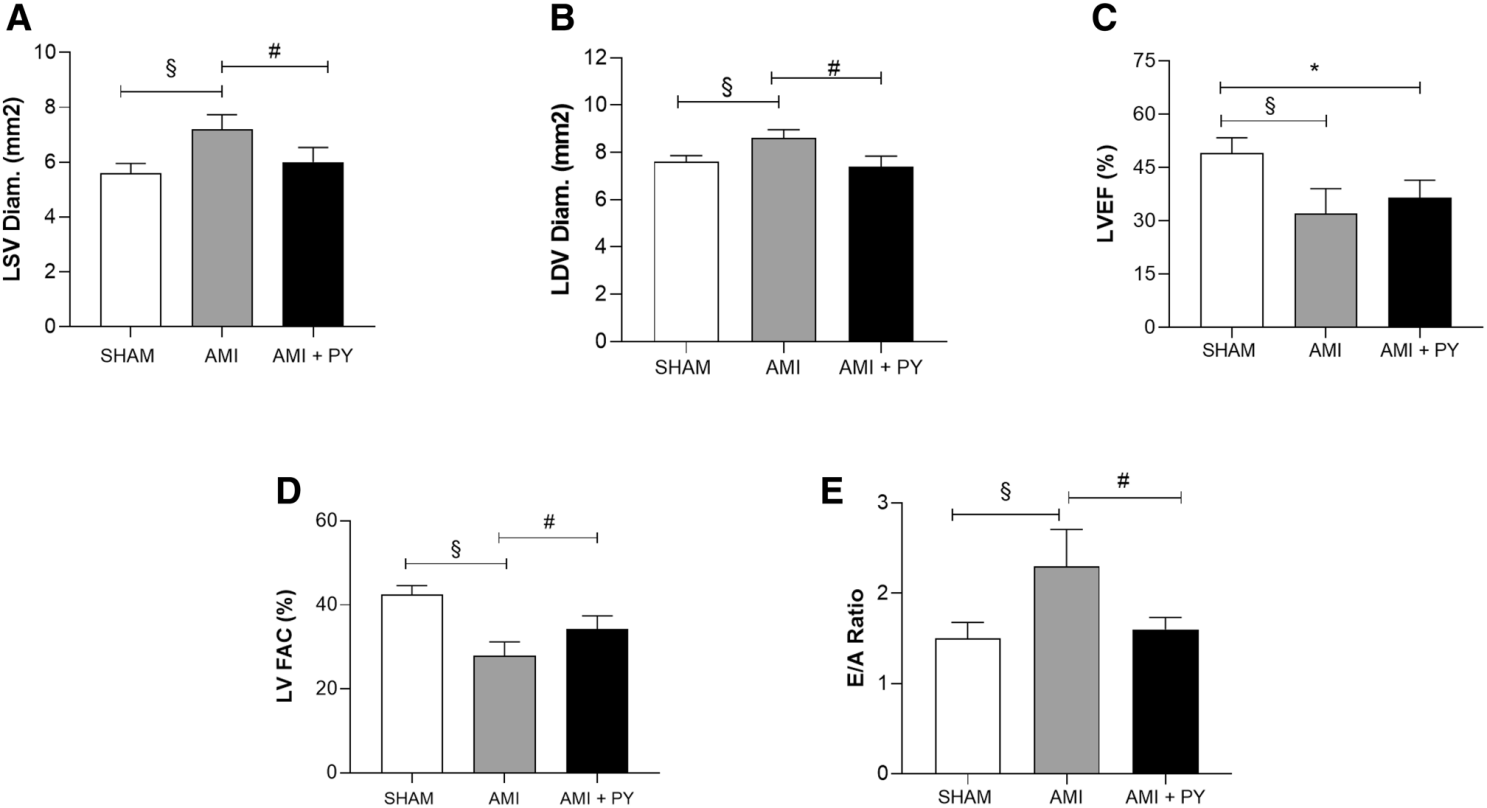
EchoDopplercardiographic parameters. (**A**) LSV Diam. And (**B**) LDV Diam.: left systolic and diastolic ventricular diameters, respectively. (**C**) LVEF: left ventricular ejection fraction (**D**) LV FAC: global left ventricular systolic function estimated by the FAC: fractional area change and (**E**) E/A: transmitral flow/E/A ratio. *P < 0.05 versus Sham; ^**#**^*P* < 0.05 versus AMI; ^**§**^*P* < 0.05 Sham versus AMI. Values expressed as mean ± SEM. Statistical significance was determined by one-way ANOVA followed by a multiple comparisons test Tukey’s was performed using GraphPad Prism version 9.0.1. www.graphpad.com.

Next, we performed structural and functional analyses to determine the effects of pyridostigmine on AMI (Table 3). AMI caused an infarcted and akinetic area consistent with moderate infarction, and significantly increased systolic and left diastolic ventricle (LV) diameters as compared to sham animals. AMI also lowered LV systolic function as inferred by LV ejection fraction (LVEF) (Fig. 3C) and fractional area change (LV FAC) (Fig. 3D), but increased E/A ratio (Fig. 3E) (early filling wave/late atrial contraction wave) that was associated with diastolic dysfunction. Together, these results indicate substantial cardiac remodeling after AMI. Pyridostigmine treatment significantly improved structural and functional parameters, but not the infarcted and akinetic area (Table 3). Pyridostigmine treatment significantly decreased systolic (LDV) and left diastolic ventricle (LSV) diameters (Fig. 3A,B, respectively). Although pyridostigmine treatment did not alter LVEF (Fig. 3C), it significantly increased fractional area change (LV FAC), which is an index of global ventricular function (Fig. 3D), and decreased the E/A ratio (Fig. 3E). These results show that pyridostigmine improved systolic and diastolic functions and cardiac remodeling after AMI in SHRs.

**Table 3:** EchoDopplercardiographic parameters in all groups.

	Sham (n = 5)	AMI (n = 6)	AMI + PY (n = 5)
% infarcted area		0.366 ± 0.03	0.350 ± 0.41
Hipo or acinetic area	–	0.172 ± 0.02	0.180 ± 0.06
AO/AE ratio (mm)	0.9 ± 0.1	0.8 ± 0.1	0.9 ± 0.1
LA Diam. (mm)	4.1 ± 0.9	4.4 ± 1.0	3.9 ± 0.6
LSV Diam. (mm^2^)	5.6 ± 0.8	7.2 ± 1.3 ^**§**^	6.0 ± 1.2 ^**#**^
LDV Diam. (mm^2^)	7.6 ± 0.6	8.6 ± 0.9 ^**§**^	7.4 ± 1.0 ^**#**^
LV Mass (g/Kg)	476.9 ± 70.3	524.9 ± 143.5	475.3 ± 171.9
LVEF (%)	49.0 ± 9.8	32.1 ± 17.2 ^**§**^	36.5 ± 11.1 *
LV FAC (%)	43.8 ± 8.6	27.2 ± 9.0 ^**§**^	35.9 ± 9.8 ^**#**^
E/A Ratio	1.5 ± 0.4	2.3 ± 1.0 ^**§**^	1.6 ± 0.3 ^**#**^
IVRT (ms)	17.8 ± 4.5	18.3 ± 4.6	20.0 ± 5.2

Values expressed as mean ± SEM. ^**§**^AMI versus Sham; AMI + PY vs Sham and ^#^AMI + PY versus AMI. LA: left atrial diameter; AO/LA Ratio: aorta/atrial diameter ratio; LSV Diam. and LDV Diam.: left systolic and diastolic ventricular diameters, respectively; LSV Vol. and LDV Vol.: left ventricular systole volume and left ventricular diastolic volume, respectively; LV mass: left ventricular mass; LVEF: left ventricular ejection fraction; LF FAC: global left ventricular systolic function estimated by the FAC: fractional area change; E: early filling wave; A: wave of late filling; E/A: transmitral flow/E/A ratio; IVRT: isovolumetric relaxation time. *P < 0.05; ^#^*P* < 0.05; ^**§**^*P* < 0.05. Statistical significance was determined by one-way ANOVA followed by a multiple comparisons test Tukey’s was performed using GraphPad Prism version 9.0.1. www.graphpad.com.

### Pyridostigmine treatment increases T helper and M2 macrophages in the infarcted and peri-infarcted zones, and inhibition of excessive release of TNF in the spleen and heart tissue

**Figure 4:**
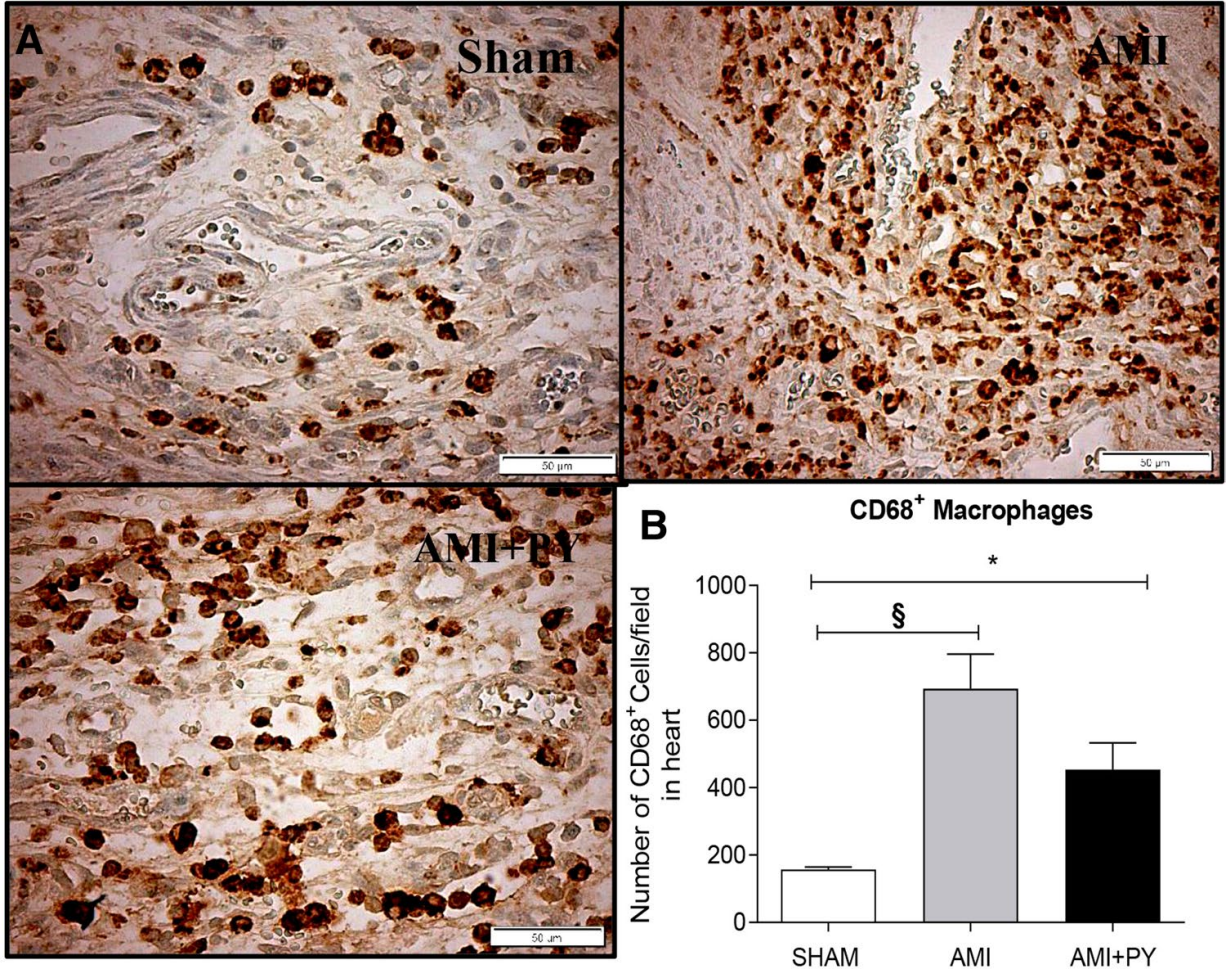
CD68^+^ cell count (M1 macrophages) (**A**) Photomicrographs showing the M1 macrophages within the infarcted zone from the left ventricle (magnification 400×) and (**B**) bar graphs of CD68^+^ positive cells in each group was compared. Fifteen microscopic fields of the infarcted and peri-infarcted zones were analyzed (5–6 animals per group). *P < 0.05; ^**§**^*P* < 0.05 statistically significant. ^**§**^AMI versus Sham; *AMI + PY vs Sham. (Immunohistochemistry, diaminobenzidine: scale bar equals 50 µm). Values expressed as mean ± SEM. Statistical significance was determined by one-way ANOVA followed by a multiple comparisons test Tukey’s was performed using GraphPad Prism version 9.0.1. www.graphpad.com.

**Figure 5:**
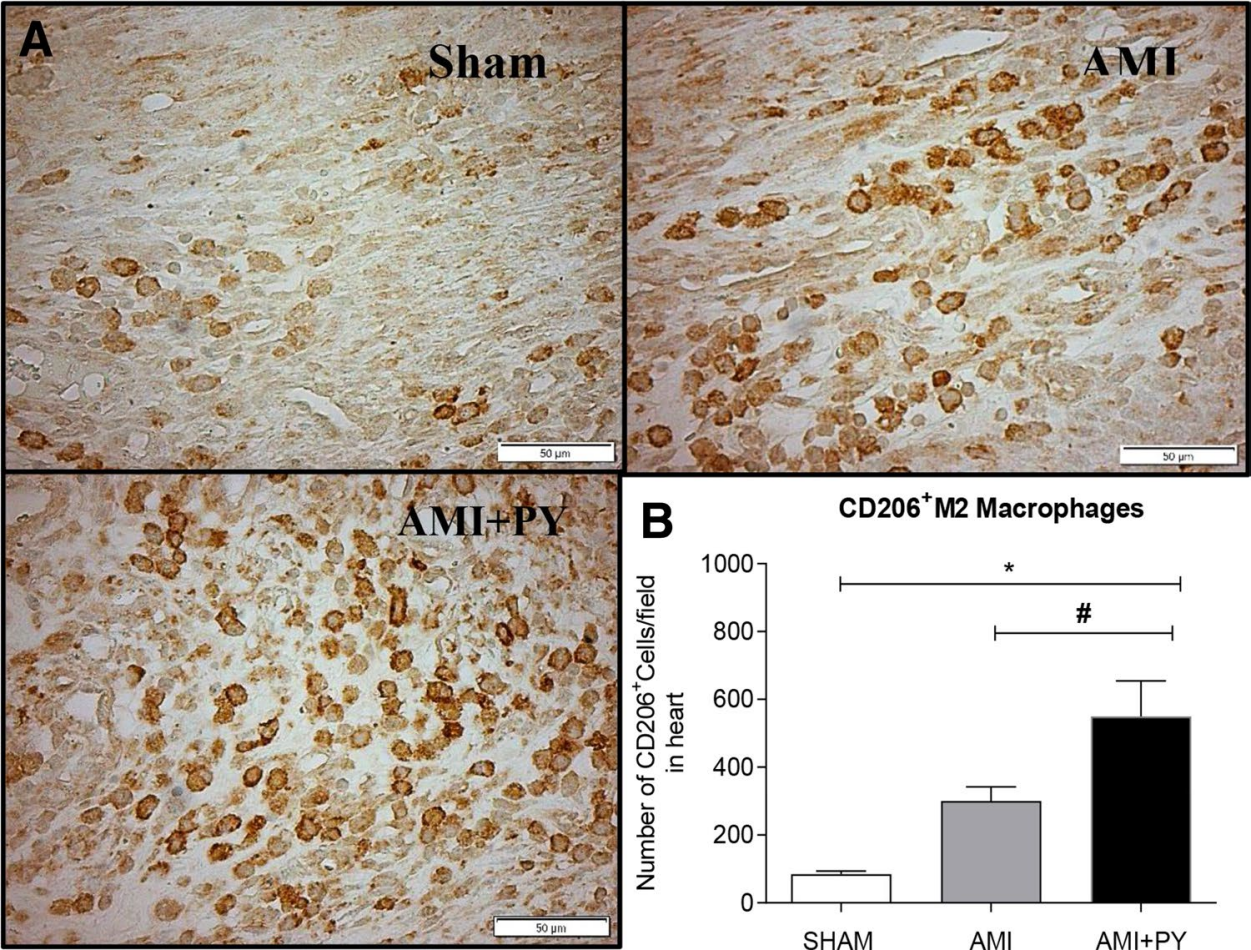
CD206^+^ cell count (M2 macrophages) (**A**) Photomicrographs showing the M2 macrophages within the infarcted zone from the left ventricle (magnification 400×) and (**B**) bar graphs of CD206^+^ positive cells in each group was compared. Fifteen microscopic fields of the infarcted and peri-infarcted zones were analyzed (5–6 animals per group). *P < 0.05; ^#^*P* < 0.05 statistically significant. *AMI + PY vs Sham; ^**#**^AMI + PY vs AMI (Immunohistochemistry, diaminobenzidine: scale bar equals 50 µm). Values expressed as mean ± SEM. Statistical significance was determined by one-way ANOVA followed by a multiple comparisons test Tukey’s was performed using GraphPad Prism version 9.0.1. www.graphpad.com.

**Figure 6:**
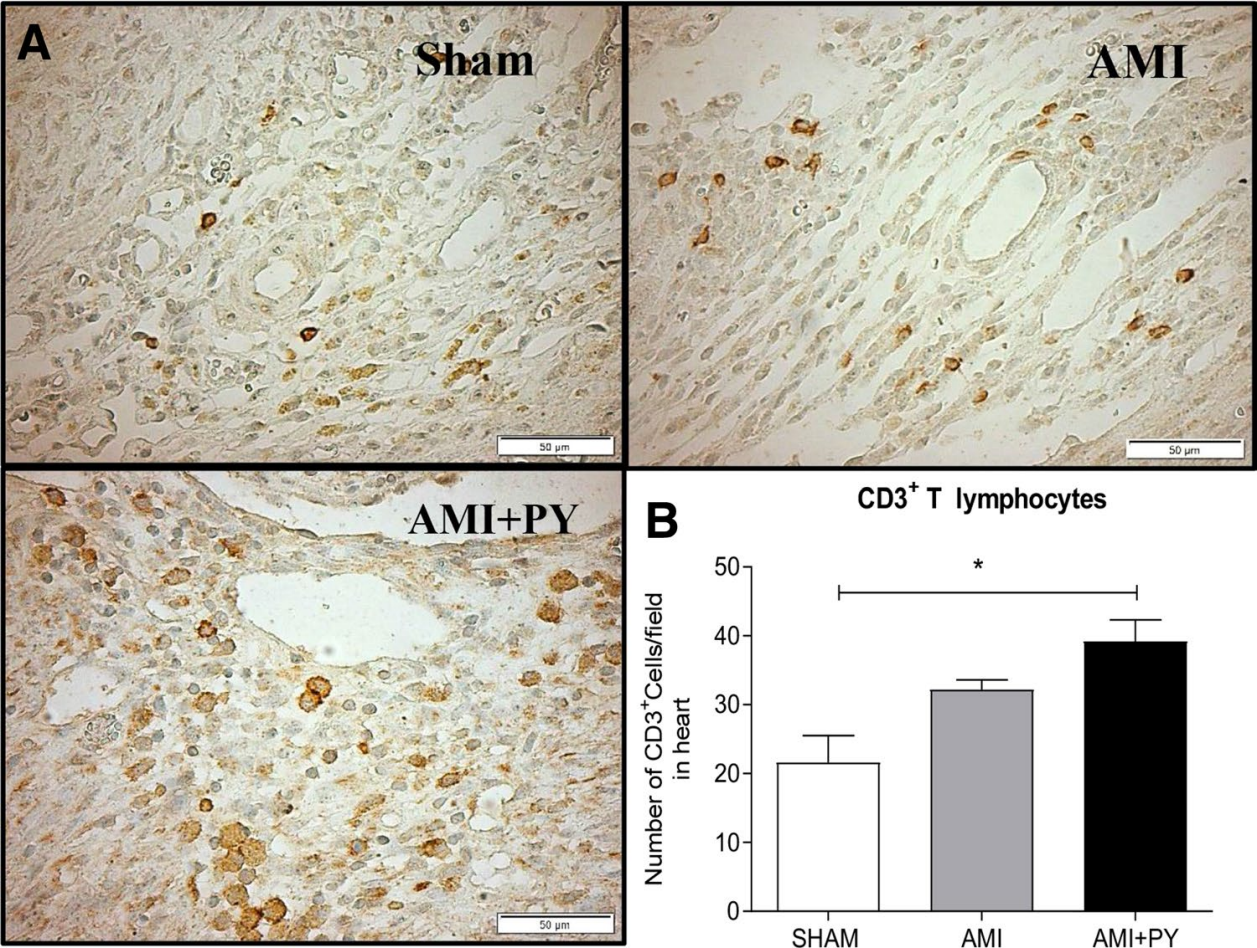
CD3^+^ cell count (Total lymphocytes) (**A**) Photomicrographs showing the Total lymphocytes within the infarcted zone from the left ventricle (magnification 400×) and (**B**) bar graphs of CD3^+^ lymphocytes positive cells in each group was compared. Fifteen microscopic fields of the infarcted and peri-infarcted zones were analyzed (5–6 animals per group). *P < 0.05 statistically significant. *AMI + PY vs Sham. (Immunohistochemistry, diaminobenzidine: scale bar equals 50 µm). Values expressed as mean ± SEM. Statistical significance was determined by one-way ANOVA followed by a multiple comparisons test Tukey’s was performed using GraphPad Prism version 9.0.1. www.graphpad.com.

**Figure 7:**
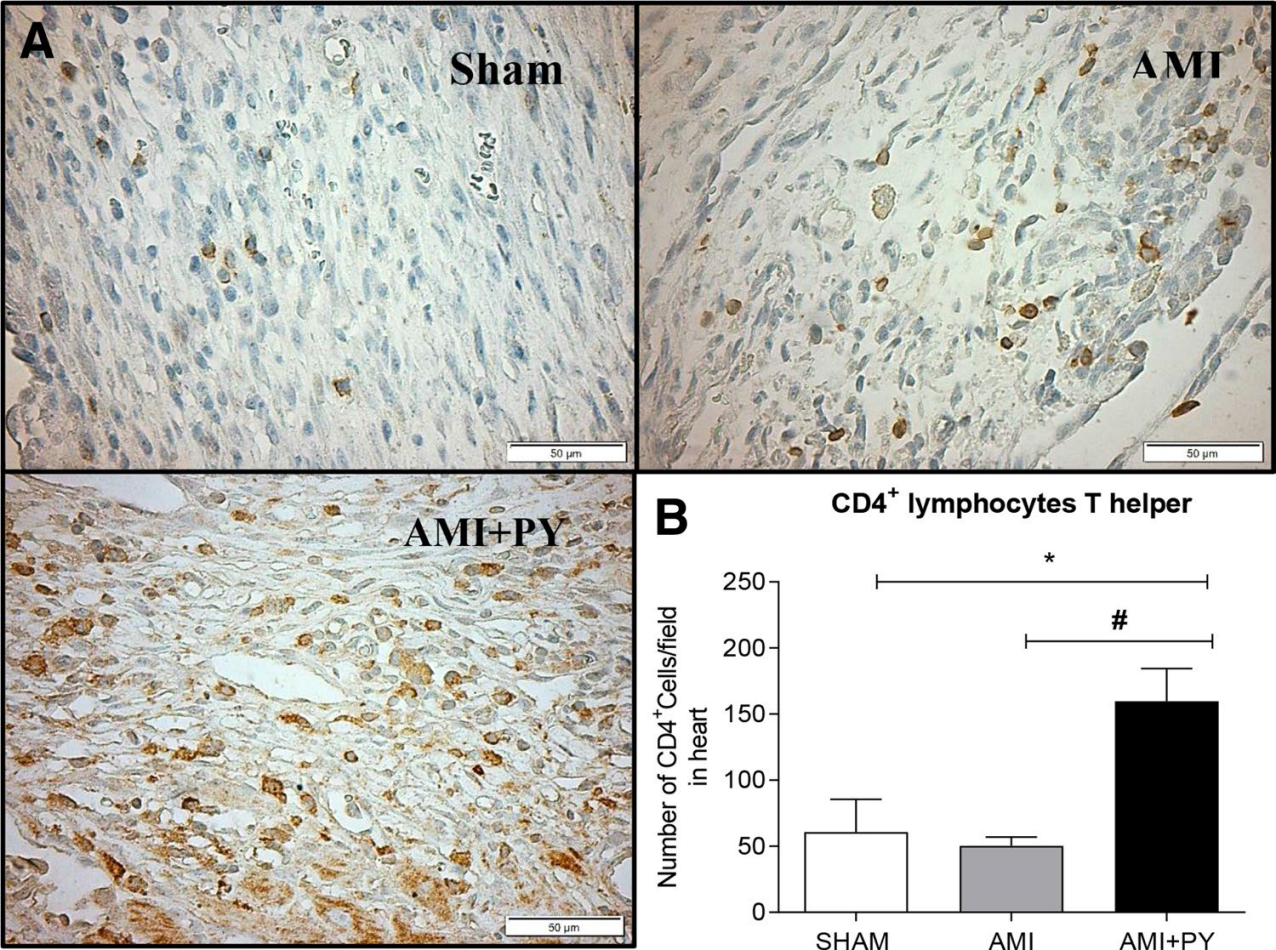
CD4^+^ cell count (Lymphocytes T helper) (**A**). Photomicrographs showing the Lymphocytes T helper within the infarcted zone from the left ventricle (magnification 400×) and **(B)** bar graphs of CD4^+^ positive cells in each group was compared. Fifteen microscopic fields of the infarcted and peri-infarcted zones were analyzed (5–6 animals per group). *P < 0,05; ^#^*P* < 0.05 statistically significant. *AMI + PY versus Sham; ^**#**^AMI + PY versus AMI. (Immunohistochemistry, diaminobenzidine: scale bar equals 50 µm). Values expressed as mean ± SEM. Statistical significance was determined by one-way ANOVA followed by a multiple comparisons test Tukey’s was performed using GraphPad Prism version 9.0.1. www.graphpad.com.

**Figure 8:**
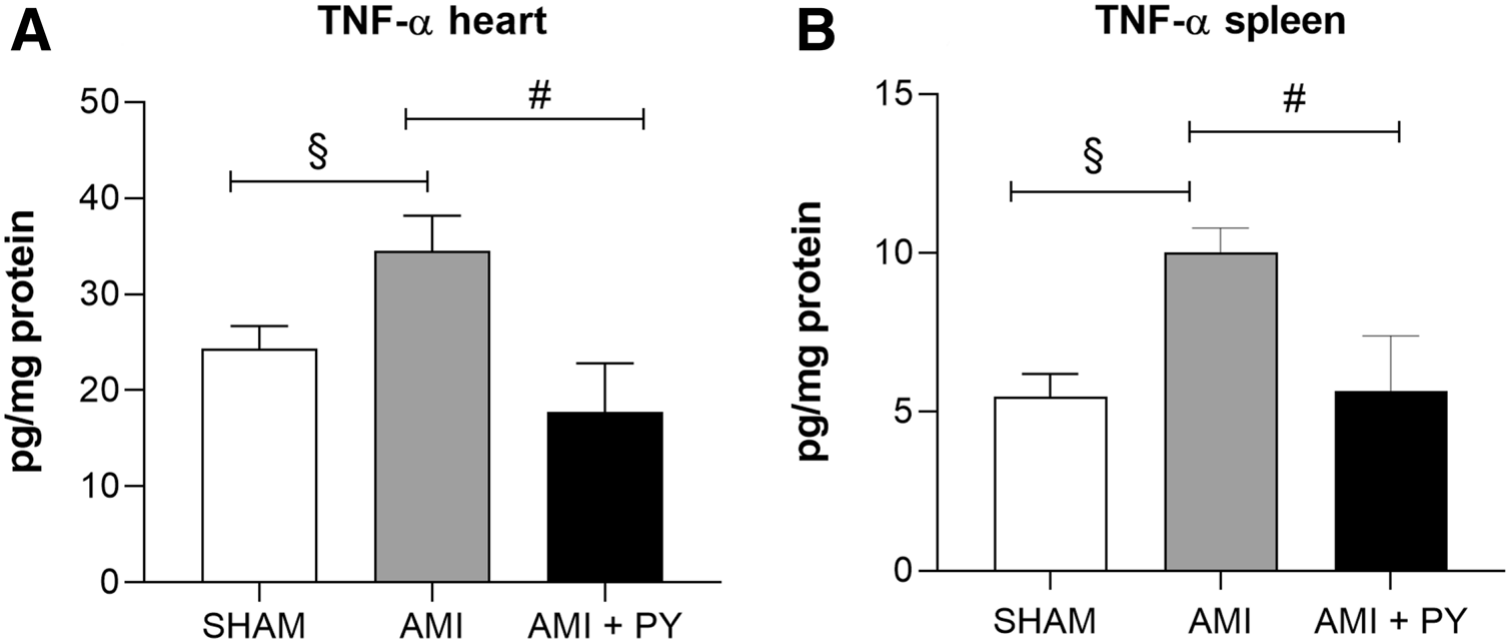
TNF levels in the heart (**A**) and the spleen (**B**) in all groups. ^#^*p* < 0.05; ^§^*P* < 0.05 statistically significant. Values expressed as mean ± SEM. One-way ANOVA. ^**§**^AMI versus Sham; ^#^AMI + PY versus AMI. Values expressed as mean ± SEM. Statistical significance was determined by one-way ANOVA followed by a multiple comparisons test Tukey’s was performed using GraphPad Prism version 9.0.1. www.graphpad.com.

Sham SRH animal have few immune cells in the myocardium. However, AMI induces a significant infiltration of immune cells into the infarcted and peri-infarcted zones at day seven. Among these cells, AMI significantly increased CD68+ (M1) macrophage counts, and pyridostigmine significantly decreased these counts (Fig. 4). Likewise, AMI also increased CD206+ (M2) macrophage counts, but pyridostigmine treatment further increased these cells in the infarcted and peri-infarcted zones (Fig. 5). Therefore, pyridostigmine treatment altered the M1/M2 ratio towards a M2 anti-inflammatory profile. We also detected that sham and AMI groups have similar counts of T (CD3 +) total lymphocytes. However, pyridostigmine treatment increased the number of T cells as compared to those in sham group, and these cells were concentrated in the infarcted and peri-infarcted areas (Fig. 6). Likewise, Sham and AMI animals had similar counts of T helper (CD4 +) lymphocytes, and pyridostigmine treatment increased these cells in the infarcted and peri-infarcted areas (Fig. 7). In agreement with previous studies, we also observed that AMI increased TNFα levels both in heart and spleen, and PY treatment significantly decreased these levels in both organs (Fig. 8A,B). Thus, AMI failed to affect the lymphocyte counts, and pyridostigmine significantly increased T helper counts, decreased the M1/M2 ratio, and decreased TNF levels in the infarcted area but also in the spleen (Fig. 8).

## Discussion

The main new findings of this study are that pyridostigmine treatment increases parasympathetic modulation and baroreflex sensitivity, promotes anti-inflammatory immune cell modulation in the myocardium, and decreases splenic and cardiac TNF levels after AMI in SHRs. These results also correlate with the potential of pyridostigmine to improve cardiac morphofunctional remodeling after AMI in SHRs. Thus, cholinergic stimulation using pyridostigmine induces cardiac protection after ischemic injury in SHRs.

The bidirectional interplay between the nervous and immune systems has gained great clinical attention to design novel therapeutic strategies for AMI^17, 33^. The inflammatory reflex and its efferent arm—the cholinergic anti-inflammatory pathway have been successfully explored in therapeutic approaches for sepsis, arthritis and other inflammatory conditions^17, 18^. Activation of cholinergic signaling in the inflammatory reflex using cholinesterase inhibitors, including the cholinergic drug galantamine suppresses inflammation in preclinical and clinical settings of numerous disorders characterized by immune and metabolic dysregulation^33, 34^. This is the first study investigating the effects of cholinergic stimulation by the cholinesterase inhibitor pyridostigmine on immune alterations after AMI in SHRs. This is a clinically relevant study, because SHRs have hemodynamic, neural, and target organ damage reminiscent of that found in essential neurogenic human hypertension. Okamoto and Aoki (1963) developed this strain of hypertensive rats^35^ that are widely used in the literature to study natural history, genetic determinants, and pathophysiological changes in arterial hypertension^36, 37^. SHRs exhibit immune deficiencies in T and B lymphocytes, decreased T cell proliferative responses^36^, and lower ChAT mRNA expression in circulating and splenic mononuclear cells as compared to normotensive Wistar rats^38^. The possibility that hypertension in SHRs is related to immune anomalies is still under investigation^36, 37^. Enhanced innate immune responses and cytokine release from splenocytes have been demonstrated in pre-hypertensive SHR rats and evoke profound activation of the adaptive immune system contributing to vascular damage and hypertension^37^. In addition, SHRs exhibit autonomic dysfunction (increased sympathetic and lower parasympathetic vagal modulation) and chronic inflammation, suggesting that they may represent an animal model that better mimics the clinical profile of patients with AMI^36, 37^.

SH animals have a higher sympathovagal ratio; AMI further increased the sympathetic drive and worsened baroreflex function. Still, pyridostigmine treatment significantly improved the cardiovascular autonomic balance and baroreflex sensitivity. The baroreflex is a fundamental self-regulatory mechanism of the cardiovascular system. La Rovere^32^ demonstrated a close correlation between post-MI mortality and baroreflex sensitivity (BRS). Although the pathological importance of impaired baroreflex function in high post-MI mortality has already been recognized, no effective long-term pharmacological intervention is available^32^, which is the direct consequence of the lack of targeted drugs. These findings suggest a mechanistic basis for restoring baroreflex sensitivity with pyridostigmine in AMI.

Pyridostigmine is a potent acetylcholinesterase inhibitor and a clinically-approved cholinergic drug for the treatment of *Myasthenia Gravis.* P*y*ridostigmine significantly increases vagal modulation as reported in experimental and human studies. Short-term administration of pyridostigmine increases HRV in healthy humans^39^ and rats^29^. In patients with heart failure, pyridostigmine also ameliorates the autonomic and hemodynamic performance during dynamic exercise^40^ and reduces ventricular arrhythmia density^41^. Also, pyridostigmine enhances cholinergic modulation of the immune cells by preventing vagal-derived acetylcholine degradation^38^. We and others have demonstrated that pyridostigmine treatment started just after coronary artery ligation can improve autonomic and cardiocirculatory function in normotensive rats analyzed weeks after the AMI^15, 16, 42^. A limited number of studies have evaluated the effects of peripheral and centrally-acting cholinesterase inhibitors on autonomic, hemodynamic, and inflammatory indices in SH animals^43, 44^. Importantly, our findings indicate that pyridostigmine induces autonomic improvement in infarcted SHRs that is associated with anti-inflammatory responses in the heart-spleen axis, and cardiac remodeling markers.

Myocardial infarction triggers a sterile inflammatory response characterized by sequential recruitment and activation of innate and adaptive immune cells to repair the tissue damage^1–3^. Macrophages are among the first responders to local damage. Depending on their phenotype, macrophages may be harmful or protective. M1 macrophages express high levels of inflammatory mediators and dominate the first days after AMI, while M2 macrophages express high levels of anti-inflammatory cytokines involved in tissue repair, gradually appear and remain predominant over five days after the infarction^1, 3^. Pyridostigmine treatment decreased M1 and increased M2 macrophages, favoring an anti-inflammatory profile with increased T helper (CD4 +) lymphocyte counts and decreased TNF levels in myocardium and spleen. These results provide new insights into the efficacy of pyridostigmine in SHRs subjected to MI and substantially extend the information generated by previous studies using normotensive infarcted animals^30, 31^. As in these previous studies, a better anti-inflammatory, immune cell profile in the myocardium was associated with decreased levels of pro-inflammatory cytokines.

The sympathetic nervous system enhances macrophage recruitment in the heart after an ischemic event. Sympathetic fibers stimulate the bone marrow to release hematopoietic stem and progenitor cells, as observed in mice and humans after AMI^6, 7, 45^. The spleen is also a major source of pro-inflammatory cytokines and immune cells. Indeed, within the first day after coronary ligation, the spleen releases monocytes from its subcapsular red pulp into the bloodstream as shown in mice and rats^6, 7, 9^. As a consequence, bone marrow and splenic leucocyte production lead to monocytosis, and these cells are recruited into the ischemic heart^8, 9, 13^. This increased metabolic activity of spleen and bone marrow has been shown in patients with acute AMI using 18F-fluorodeoxyglucose PET image^46^. Our results show that pyridostigmine decreased sympathovagal balance in infarcted rats, improved M1/M2 macrophage ratio, and decreased TNF levels in spleen and heart. Thus, cholinergic stimulation may interfere with local mechanisms preventing macrophage polarization in the ischemic myocardium. The idea of targeting macrophage and lymphocyte polarization to control inflammation following AMI has been recently proposed^47^. In addition to innate cells, lymphocytes also play a fundamental role after AMI^10^. More specifically, CD4+ T helper cells are required for proper healing and can attenuate chronic remodeling after AMI. CD4+ T-cells regulate the infiltration of inflammatory monocytes and are required for proper extracellular matrix formation and angiogenesis during post-MI healing^48^. Furthermore, CD4+ T cell-derived cytokines modulate monocyte differentiation and macrophage activity within the myocardium^49^.

Our results indicate a link between increased vagal modulation with pyridostigmine and an anti-inflammatory M1/M2 profile, increased T helper counts, and decreased myocardium TNF levels. Given the detrimental potential of TNF on the myocardium, TNF local production may play an important role in ventricular dysfunction and adverse remodeling after infarction^50^. TNF is produced soon after AMI, regulates myocardial apoptosis and triggers additional cellular inflammatory responses^50^. Anti-TNF treatment was associated with smaller infarct size and decreased ventricular dysfunction in ischemic-reperfusion and permanent ischemia in animal models^51^ including AMI. Thus, it is plausible that TNF inhibition by pyridostigmine may trigger a positive remodeling and decrease heart failure in infarcted SHRs.

Targeting cholinergic signaling in the inflammatory reflex provides novel therapeutic opportunities for diseases characterized with inflammatory and cardiovascular derangements, including myocardial ischemia–reperfusion injury and AMI damage^17^. The vagus nerve can act reflexively on the spleen, reducing systemic inflammation^18^. Likewise, efferent vagal signals may facilitate the release of lymphocytes from the thymus through nicotinic receptors^52^. Vagal stimulation can increase acetylcholine release, which activates α7nAChR on macrophages to decrease pro-inflammatory cytokines in the spleen^19,22,24^. A main source of acetylcholine in the spleen is a subgroup of T lymphocytes expressing β2-adrenoreceptors and ChAT (ChAT-T cells) that synthesize acetylcholine^22^. Upon vagal activation, the splenic nerve releases norepinephrine (NE) in the spleen to stimulate ChAT-T cells to release acetylcholine, which in turn inhibits TNF production in macrophages via α7nAChR-mediated signaling^15, 20, 21, 23^.

As both acetylcholine and NE modulate splenic immune cell functions, our study highlights the need to consider the balance between these two mediators, which probably determines the final immunoregulatory outcome^53^. We show the potential of pyridostigmine to reduce cardiac inflammation and improve heart remodeling after AMI. As expected, AMI caused significant morphofunctional alterations, detected by echocardiography at day 7, which affects circulation and reduces blood pressure. Pyridostigmine treatment significantly improves systolic and diastolic function, but they were insufficient to restore normal blood pressure as seen in sham animals. Noninvasive diastolic function measurements with echocardiography correlated with LV cardiac measurements as determined by catheterization in rat models of myocardial hypertrophy and AMI^22^; these noninvasive parameters may predict further ventricular dysfunction^5^. Ventricular dysfunction and heart failure are frequent complications of AMI and are associated with poor prognosis. Some studies have shown the beneficial effects of more prolonged pyridostigmine treatment on cardiac function and remodeling in normotensive infarcted rats^15, 54, 55^. It can be speculated that this immediate improvement may lead to positive remodeling and decrease the development of heart failure in infarcted SHRs.

Our study has some limitations. As mentioned above, the specific role of ChAT-positive T cells and possibly B cells in the effects observed in the spleen is unknown and remains to be evaluated in future studies. In addition, there is a lack of specific insight into the role of ChAT-containing immune cells in the heart and cardiac myocytes in mediating local immunomodulatory effects in our model. On a related note, we cannot assess the relative contribution of neural acetylcholine versus non-neuronal acetylcholine in the beneficial immune and cardiometabolic pyridostigmine effects on the heart. Previously, vagus nerve stimulation has been shown to suppress cardiac (myocardial) and systemic TNF levels in rodents during endotoxemia^18, 19^. Vagus nerve stimulation in animals and patients with heart failure also results in improved cardiac function, indicating the cardioprotective efficacy of vagus nerve cholinergic signaling^56–58^ Previous studies have indicated that cardiac myocytes also contain ChAT and synthesize and secrete acetylcholine, which plays a role in myocardium regulatory functions^59, 60^. Acetylcholine released from cardiac myocytes reportedly has various protective effects in pathological scenarios, including cardiac hypertrophy and failure as indicated in transgenic mouse models^61, 62^. Further studies are necessary to evaluate the specific role of non-neuronal cell-derived acetylcholine in addition to the neural vagus nerve-derived cholinergic output in mediating pyridostigmine effects in the heart. In addition, further work using cell-type-specific transcriptomic and epigenomic analysis is necessary to reveal the underlying molecular mechanisms of how pyridostigmine induces the M1-M2 cell type conversion.

In conclusion, cholinergic stimulation with pyridostigmine modulates a heart-spleen axis in the immune response after myocardial infarction and improves heart remodeling. Our results warrant future studies to determine mechanisms of these effects and the consequences in heart failure.

## Materials and methods

### Experimental design

All animal procedures were approved by the Committee on the Ethics of Animal Experimentation at the University of Nove de Julho (CEUA protocol No. 7612011118). Animal care was performed following the *Guide for the Care and Use of Laboratory Animals* published by the U.S. National Institutes of Health. Adult male SHR (2–3 months old, 200–250 g) were housed in collective plastic cages (four animals per cage) with controlled temperature (23 °C), a 12:12-h light–dark cycle with rat chow provided ad libitum and unlimited access to water. Animals were randomly assigned to one of three groups, with 10–12 animals in each group: sham rats (Sham), untreated infarcted rats (AMI), and pyridostigmine-treated infarcted rats (AMI + PY). All animals were monitored for 7 days. The AMI + PY Group received pyridostigmine bromide (Sigma-Aldrich, St Louis, MO), as described previously (40 mg/kg once a day, by gavage) started one h after surgery and continued for seven days after this procedure^8, 63^. According to a prior study, the dose and period of pyridostigmine administration chose were appropriate to inhibit approximately 40% of plasma acetylcholinesterase activity^29^. All methods were reported in accordance with ARRIVE guidelines.

### Myocardial infarction

Rats in the AMI and AMI + PY groups were anesthetized (80 mg/kg ketamine and 12 mg/kg xylazine intraperitoneal injected, I.P.) and underwent induction of AMI by surgical occlusion of the left coronary artery, as previously described^30, 31^. A left thoracotomy performed by dissecting the third intercostal space and exposing the heart. Then, the left coronary artery was occluded with a single nylon (6.0 mm) suture 1 mm distal to the left atrial appendage. The chest was then sutured. The rats were maintained under ventilation until recovery. The sham group underwent the same procedure, but AMI was not induced. Infarcted rats were randomly allocated to receive or not P.Y. The analytical investigators were blind to the treatment.

### Arterial catheterization, hemodynamic measurements, and cardiovascular variability analysis

At day six, rats were anesthetized (80 mg/kg ketamine and 12 mg/kg xylazine, I.P.), and a catheter filled with 0.06 mL of saline solution was implanted into the femoral artery^29^ for hemodynamic measurements. The arterial cannula was connected to a strain gauge transducer (Blood Pressure XDCR; Kent Scientific, Torrington, CT), and arterial pressure (A.P.) signals and pulse interval heart rate (H.R.) were digitally recorded over a 30-min period in conscious, awake animals using a data acquisition system (WinDaq, 2 kHz; DATAQ, Springfield, OH), as described^29^. This basal acquisition was used to evaluate heart rate variability (HRV) and systolic arterial pressure variability (as described below). *HRV.* For time and frequency domains analysis of cardiovascular autonomic modulation, the time series (three time series of 5 Min for each animal) of pulse interval (PI) and systolic arterial pressure (SAP) were cubic spline-interpolated (250 Hz) and cubic spline-decimated to be equally spaced in time after linear trend removal; power spectral density was obtained through the Fast Fourier transformation. Spectral power for low frequency (LF, 0.20–0.75 Hz) and high frequency (HF, 0.75–4.0 Hz) bands was calculated by power spectrum density integration within each frequency bandwidth, using a customized routine (Cardioseries). The time domain variables were: root mean square of the successive differences (RMSSD) and total variance of pulse interval (VAR-PI) for pulse interval (PI); and total variance of systolic arterial pressure (VAR-SAP) for systolic arterial pressure (SAP). The α-index in the low-frequency band was calculated only when the magnitude of the squared coherence between the PI and SAP signals exceeded 0.5 (range, 0–1). After coherence calculation, the α-index was obtained from the square root of the ratio between PI and SAP variability in the two major low frequency (LF) band^29^.

### Echocardiographic evaluation

Echocardiographic evaluations were performed by a blind observer under the guidelines of the American Society of Echocardiography. Rats were anesthetized (80 mg/kg ketamine and 12 mg/kg xylazine intraperitoneal-I.P.), and images were obtained with a 10–14-MHz linear transducer in a G.E. *Vivid 7* Ultra-Definition Clarity Control (G.E. Healthcare, USA). This procedure was performed six days after AMI or sham surgeries in order to analyze AMI area (hipo or acinetic ventricular areas) and LV ejection fraction (LVEF%), and to calculate the following parameters: left atrial diameter, ventricular mass (LV mass); left ventricular end-diameter during systole and diastole (LVSD, LVDD); E wave A wave ratio (E/A); isovolumetric relaxation time (IVRT); fractional area change (FAC), as described in detail elsewhere as described previously^15, 63^. Through midtransversal and apical transversal views, AMI size was measured by bi-dimensional echocardiogram. In diastole, three measurements of the endocardial perimeter (E.P.) and the length of the infarcted segment (ISe) were obtained for each view. The AMI size for each ISi was calculated by the equation ISi (%) = ISe/E.P. × 100. The total infarct size of each animal was calculated as the mean of ISi (%) of the three segments. AMI was defined as increased echogenicity and change in myocardial systolic movement (hypokinesia, akinesia, or dyskinesia), following Santos et al.^28^. Our group^15^ and others^63^ have demonstrated strong correlations between the AMI area assessed by echocardiogram and *postmortem* histological analysis, showing that this is a valid method to estimate AMI area in rats.

### Immunohistochemistry for immune cells

At day seven, 6–7 animals from each group were anesthetized (80 mg/kg ketamine and 12 mg/kg xylazine, I.P.) and perfused with 0.9% NaCl plus 14 mmol/l KCl solution (IV; with a pressure equal to 13 cmH_2_O) to arrest the heart in diastole, followed by perfusion of 4% buffered formalin for tissue fixation. Harvested hearts were immersed in formalin for 24 h. Transverse slices were processed and embedded in paraffin. Serial sections of paraffin-embedded tissues (3 μm) were placed on glass slides coated with 2% 3-aminopropyl-triethyl silane (Sigma-Aldrich) and deparaffinized in xylene, then immersed in alcohol and incubated with 3% hydrogen peroxide. The sections were immersed in a citrate buffer (pH 6.0; Sigma- Aldrich) at 95 °C for 20 min for antigen retrieval. Nonspecific signals were blocked using specific antibody diluents (Antibody Diluent, cat. no. S0809; Dako, Glostrup, Denmark)^30^. The slides were then incubated with the following primary antibodies: anti-rat CD4 (1:500, T Lymphocyte marker, rabbit monoclonal—EPR19514, cat. no. 221775; Abcam, Cambridge, UK), anti-rat CD3 (1:100, T helper Lymphocyte marker, rabbit monoclonal—SP7; cat 16669, Abcam), anti-rat CD68 (1:100, M1 macrophage marker, mouse monoclonal—ED-1 cat. no. 31630, Abcam), and anti-rat CD206 (1:800, M2 macrophage marker cat. no. 64693; Abcam). The samples were kept overnight at 4 °C in a humidified chamber. The sections were incubated with LSAB + System-HRP reagents for 30 min (K0690; Dako Co, Denmark). Finally, the sections were incubated in 3,3-diminobenzidine in a chromogen solution (K346811; Dako Co, Denmark) at room temperature for 2–5 min, and then stained with Mayer's hematoxylin (Sigma-Aldrich) and covered. For the negative controls, the primary antibodies were replaced with 1% PBS/BSA and nonimmune mouse serum (X501-1, Dako).

### Cell counts in the infarcted and peri-infarcted zones

Fifteen consecutive microscope fields (magnification: 400×) of the infarcted and peri-infarcted zones were photographed (fluorescence Microscope (Olympus AX70) with a digital camera (Olympus Japan Co, Tokyo, Japan). An investigator blinded to the animal group samples analyzed the images and manually counted them with the aid of the Image J version 1.48v 17 (free software, NIH, Bethesda, Maryland, EUA), using the "cell counter" plug-in^31^.

### Cytokine measurements

A set of 5–6 animals in each group was euthanized by decapitation on day seven after thoracotomy to collect fresh heart and spleen for TNF-α analyses. Measurement of the TNFα was performed in samples of the LV and spleen by ELISA using Duo-set available kits for TNFα (BD Pharmingen, San Jose, CA, USA) as previously describe^30^. The sensitivity of the assays was 15 pg/mL. The results were normalized by LV or spleen total protein^30^.

### Statistical analysis

All data were represented as means ± the standard error of the mean (SEM). For parametric data, the one-way analysis of variance (one-way ANOVA) was performed with Turkey's multiple comparison tests was performed using GraphPad Prism version 9.0.1 for Windows, GraphPad Software, San Diego, California USA, www.graphpad.com. For nonparametric data, the Kruskal–Wallis test was used. *P* values less than 0.05 were considered significant.

## Accordance statement

All methods were reported in accordance with ARRIVE guidelines.
